# An RNA replication-center assay for high content image-based quantifications of human rhinovirus and coxsackievirus infections

**DOI:** 10.1186/1743-422X-7-264

**Published:** 2010-10-11

**Authors:** Andreas Jurgeit, Stefan Moese, Pascal Roulin, Alexander Dorsch, Mark Lötzerich, Wai-Ming Lee, Urs F Greber

**Affiliations:** 1 Institute of Molecular Life Sciences, University of Zurich, Winterthurerstrasse 190, CH-8057 Zurich, Switzerland; 2 Molecular Life Sciences Graduate School, ETH and University of Zurich, Switzerland; 3 3-V Biosciences GmbH, Schlieren, Switzerland & Menlo Park, CA, USA; 4 Department of Pediatrics, School of Medicine and Public Health, University of Wisconsin, Madison, Wisconsin, USA

## Abstract

**Background:**

Picornaviruses are common human and animal pathogens, including polio and rhinoviruses of the enterovirus family, and hepatits A or food-and-mouth disease viruses. There are no effective countermeasures against the vast majority of picornaviruses, with the exception of polio and hepatitis A vaccines. Human rhinoviruses (HRV) are the most prevalent picornaviruses comprising more than one hundred serotypes. The existing and also emerging HRVs pose severe health risks for patients with asthma or chronic obstructive pulmonary disease. Here, we developed a serotype-independent infection assay using a commercially available mouse monoclonal antibody (mabJ2) detecting double-strand RNA.

**Results:**

Immunocytochemical staining for RNA replication centers using mabJ2 identified cells that were infected with either HRV1A, 2, 14, 16, 37 or coxsackievirus (CV) B3, B4 or A21. MabJ2 labeled-cells were immunocytochemically positive for newly synthesized viral capsid proteins from HRV1A, 14, 16, 37 or CVB3, 4. We optimized the procedure for detection of virus replication in settings for high content screening with automated fluorescence microscopy and single cell analysis. Our data show that the infection signal was dependent on multiplicity, time and temperature of infection, and the mabJ2-positive cell numbers correlated with viral titres determined in single step growth curves. The mabJ2 infection assay was adapted to determine the efficacy of anti-viral compounds and small interfering RNAs (siRNAs) blocking enterovirus infections.

**Conclusions:**

We report a broadly applicable, rapid protocol to measure infection of cultured cells with enteroviruses at single cell resolution. This assay can be applied to a wide range of plus-sense RNA viruses, and hence allows comparative studies of viral infection biology without dedicated reagents or procedures. This protocol also allows to directly compare results from small compound or siRNA infection screens for different serotypes without the risk of assay specific artifacts.

## Background

The family of picornaviridae comprises a wide variety of human and animal pathogens [1]. Notable members of the twelve genera are the enteroviruses, such as poliovirus, the causative agent for poliomyelitis, which affected millions of people before broad vaccinations became available in the last decades. Within the picornavirus subgenera, the number of serotypes per species varies from three in the case of poliovirus up to more than one hundred for human rhinoviruses (HRV). HRVs are the main cause of common cold [2], and for recurring infections in humans [3]. HRV infections lead to severe exacerbations in patients with asthma or chronic obstructive pulmonary disease [4]. HRVs comprise species A, B and C [2]. Twelve HRVs from species A bind to the minor receptors from the low density lipoprotein (LDL) receptor family, and the other 61 A-members as well as the B-viruses bind to intercellular adhesion molecule 1 (ICAM-1) for infection [5]. The receptor(s) for the HRV-C serotypes are unknown. The enterotropic coxsackieviruses (CV) can cause myocarditis, pancreatitis and meningitis. The hepatitis A hepatovirus is responsible for mild forms of human hepatitis. An example of a non-human picornavirus is the foot-and-mouth disease virus of the apthovirus genus, which induces lesions in cloven-hoof animals, such as cattle, swine, goat, sheep and buffalo, and is the cause for tremendous economic losses, as experienced during the last outbreak in England in 2001 [6].

Picornaviruses are small, non-enveloped RNA viruses with an icosahedral capsid of about 28-30 nm in diameter [7], and a single strand, plus-sense RNA genome, which is in case of enteroviruses about 7.2 to 8.45 kb [8]. The genome encodes a single polyprotein that is proteolytically processed by viral proteases into structural and non-structural proteins. The replication of picornaviruses takes place in the cytoplasm in close association with endo-membranes containing single-and multi-membrane vesicles and complex membranous structures of various sizes [9]. Cytoplasmic membranes are well known to support the replication of plus-sense RNA viruses, for example the alphavirus Semliki Forest virus [10-12], the rubivirus rubella virus [13,14], the enterovirus poliovirus [15], or the flaviviruses hepatitis C, Dengue and West Nile viruses [16-18], where it is referred to as *membranous web*. Membrane associated replication structures are thought to protect the replicating viral RNA from anti-viral factors recognizing double-strand RNA (dsRNA), and may provide a scaffold for anchoring and locally concentrating the viral replication complexes. Since its establishment requires *de novo *lipid synthesis, it may represent an anti-viral target, as suggested from work with drosophila C virus, a dicistronic virus, which is in many ways similar to picornaviruses, for example, encoding a polyprotein by a single positive-strand RNA genome, or using cap-independent, internal ribosome entry site-dependent translation [19,20].

The replication process of viruses has been a target for classical anti-viral agents directed against proteases, polymerases or integrases in the case of human immunodeficiency syndrome viruses (HIV) or hepatitis C viruses (HCV) [reviewed in [21]]. Enterovirus inhibitors have been developed against the HRV protease 3C [22] or the capsid uncoating mechanism [for example, pleconaril, [23]]. Alternative approaches against host factors that support viral replication included protein kinases involved in virus entry, such as the serine/threonine kinase PAK1 for echoviruses, adenoviruses or vaccinia virus [24-28], as well as tyrosine kinases for coxsackievirus B3-RD [29] or microbial pathogens [for a review, see [30]]. To enhance the identification of anti-viral agents, standardized infection assays should be developed for cultured cells as a basis for high throughput screening projects.

Here we describe a simple immunofluorescence-based infection protocol to quantitatively assess infection of cultured cells with enteroviruses, using the mouse monoclonal anti-dsRNA antibody J2 [mabJ2, [31]]. It recognizes dsRNA duplexes larger than about 40 bp and was used earlier to detect replicating HCV genomes in distinct cytoplasmic foci [32], or RNA replication intermediates from the groundnut rosette virus RNA-dependent RNA polymerase [31]. The cytoplasmic foci recognized by mabJ2 are similar to foci recognized by an anti-dsRNA serum in rubella virus or Semliki Forest virus-infected cells [13,33]. We found that the appearance of mabJ2-positive dsRNA replication centers in HRV or coxsackievirus infected cells correlated with the emergence of capsid protein epitopes and infectious virus titer, and the mabJ2 assay was applicable for prototypic high throughput, image-based siRNA and small compound screens.

## Results

### Double-strand RNA replication centers identify HRV and coxsackievirus infected cells

**Figure 1:**
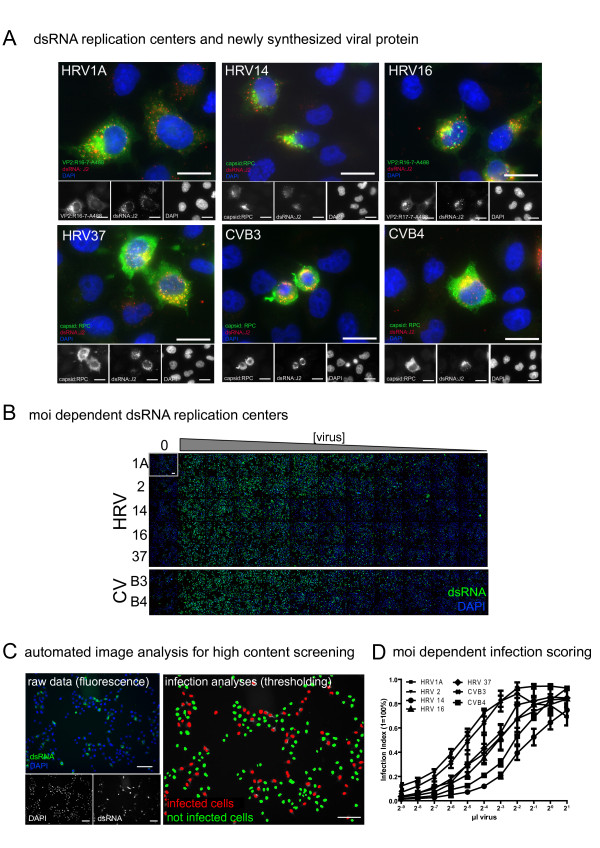
**MabJ2 detects viral replication-induced dsRNA in high content image based assays**. (A) Cells with dsRNA replication centers are positive for newly synthesized viral protein. HeLa cells were infected with the indicated HRV or CV serotypes, fixed and stained with mabJ2 (red) or capsid specific antibodies (green). CVB3, CVB4, HRV37 and HRV14 were stained with a rabbit polyclonal serum (rpc); HRV1A and 16 were stained by mabR16-7 covalently labelled with Alexa488 (R16-7-488). Magnification 60×; scale bar 20 μm. (B) Appearance of dsRNA replication centers is moi dependent. Example overview of a 96 multiwell plate of HeLa cells infected with serial dilutions of indicated HRV or CV serotypes. Imaging by automated microscopy was with 10× magnification. One out of nine images per well is shown for each condition. dsRNA replication centers (green) and DAPI stained nuclei (blue) are shown. Scale bar 100 μm. (C) An example for automated fluorescence image analysis to score infection of HeLa cells with HRV16 (moi 0.3) with raw images on the left and an image processed and pseudocolored with a Matlab algorithm on the right side. Scale bar 100 μm. (D) Example for the quantification of moi dependent fraction of infected cells (infection index) of the experiment shown in (B), and analysis by the scoring algorithm presented in (C). More detailed characterisations (time, dose) of this assay are shown in the subsequent figures.

We first tested if the formation of dsRNA-positive replication centers can be used as an assay for infection of HeLa cells strain Ohio (herein referred to as HeLa) with HRV or CV. HeLa cells are widely used to isolate and study HRVs and other enteroviruses [34]. Cells were infected at low multiplicity of infection (moi 0.2-0.4) with HRV1A, 14, 16, 37 or CVB3 or B4, and co-stained by double label immunofluorescence for dsRNA using mabJ2, and newly synthesized viral proteins using mabR16-7-Alexa488 (conjugated with Alexa488 dye) or a rabbit polyclonal antibody raised against purified capsid proteins (Fig. 1A). MabR16-7 had been raised against HRV16 and recognized VP2 from both HRV16 and 1A [35]. As expected, all cells positive for newly synthesized viral protein were also positive for dsRNA detected by mabJ2, and replication foci had a subcellular localization similar to cytoplasmic foci, which had been reported earlier as replication centers in picornavirus-infected cells [15,36]. Performing a similar experiment with the mabK1, detecting dsRNA >40bp, gave identical results, although with lower signal intensity (data not shown). We hence used mabJ2 for all following experiments.

Attempts to detect incoming viral particles by mabJ2 failed, although incoming HRV16 have been successfully visualized with mabR16-7, detecting a capsid epitope (data not shown). This was in agreement with the notion that mabJ2 detects long duplexes of double-strand structures of the replicating RNA rather than genomic RNA, that is, most likely duplexes of postive and negative-strand RNAs [31,32]. Biochemical assays estimated the numbers of negative-strand RNA copies in poliovirus infected HeLa cells to about 1000 per cell at the log phase of replication, corresponding to a few percent of the total viral RNA [37]. Since poliovirus replicates to higher levels than HRV in HeLa cells as determined, for example, in single step growth curves (WML, unpublished), we suggest that our image-based assay detects less than 1000 dsRNA molecules per cell. Although it might be possible to correlate the mabJ2 signal intensity with the viral RNA load per cell, this would require higher resolution image acquisition and quantitative measurements, and hence would reduce the throughput of the assay, and require orders of magnitude more data to be processed, which would limit the utility of this assay for screening purposes.

To test if the mabJ2 assay is useful for high-content, image-based infection screens, we infected HeLa cells with serial dilutions of different HRV and CV serotypes in multiwell plates, followed by staining with mabJ2 and counterstaining of the cell nuclei with 4′,6'-diamidin-2-phenylindol (DAPI, Fig. 1B). Non-infected cells did not show detectable signals from mabJ2, while cells inoculated with HRV1A, 2, 14, 16, 37 or CVB3 or B4 showed dose-dependent mabJ2 signals. Infected cells were quantified using a custom-written Matlab routine. This algorithm scored cells as infected, if the DAPI signal overlapped with a thresholded infection marker, which were either the newly synthesized viral protein or dsRNA replication centers (Fig. 1C, and additional file 1, Fig. S1). This analysis did not discriminate between "weak" and "intense" infection signals, but rather scored cells as *infected *if certain criteria were met (see details described in the methods section and additional file 1, Fig. S1). The analysis confirmed that the mabJ2 infection assay was robust and specific for HRV1A, 2, 14, 16 and CVB3, B4 infections in a dose-dependent manner (Fig. 1D).

**Figure 2:**
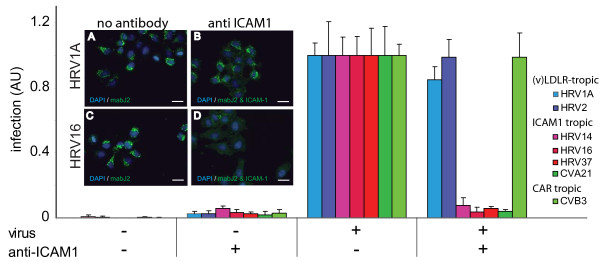
**ICAM-1 receptor blocking antibodies abolish the formation of dsRNA replication centers by major group HRVs and CVA21**. HeLa cells were pre-incubated with anti-ICAM-1 mab15.2L for 30 min and infected with indicated HRV and CV serotypes. Infection was quantified by the mabJ2 anti-dsRNA antibody using automated image acquisition and analysis. Fold infections relative to untreated control cells are indicated in arbitrary units (AU). The means including standard errors of the mean (SEM) from four independent infections are shown. Example images for HRV1A (A, B) and HRV16 (C, D) are shown, scale bar 25 μm.

For a biological validation of the mabJ2 assay, we performed a receptor interference experiment using the mouse monoclonal antibody mab15.2L to block the binding site of major HRV serotypes 14, 16 and 37 and CVA21 on the intracellular adhesion molecule 1 (ICAM-1) [38-40]. As expected, for ICAM-1 tropic HRVs and CVA21, receptor blocking led to a >90% decrease of infection, whereas minor group HRVs and CVB3, which use the low density lipoprotein (LDL)-receptor or coxsackievirus adenovirus receptor (CAR), respectively [41,42], were not affected (Fig. 2). Note that a low amount of mabJ2 signal (approximately 5%) was detected in non-infected cells treated with the mouse anti-ICAM-1 antibody, but not in non-antibody treated cells, and hence represents the reactivity of the secondary anti-mouse antibody (see additional file 2, Fig. S2). We conclude that the mabJ2 replication center assay is reliable and has a good signal-to-noise ratio.

### Towards high content image based infection screening

**Figure 3:**
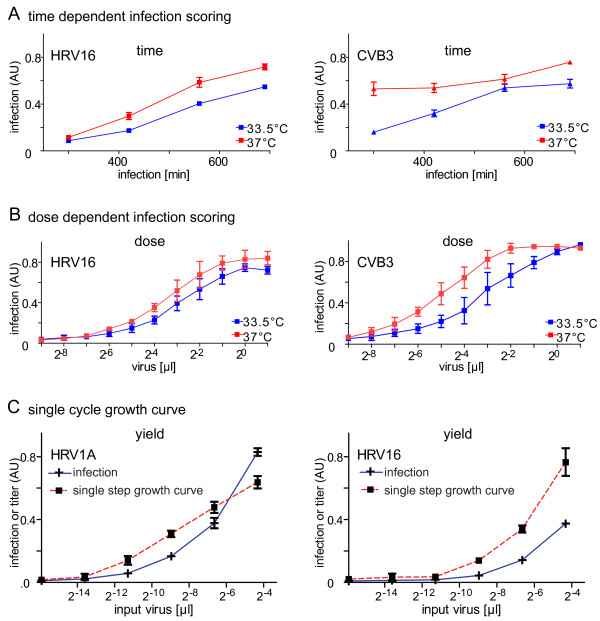
**Appearance of dsRNA replication centers is time, dose and temperature dependent and correlates with emergence of infectious titres**. (A, B) The time and dose dependencies of HRV16 and CVB3 infections at 33.5°C (blue) or 37°C (red) were determined using the mabJ2 dsRNA infection assay in HeLa cells by either infection for 300 to 700 min, or with two fold serial dilutions of inocula. (C) To determine the correlation of mabJ2 dsRNA staining with viral titre production, HeLa cells were infected with HRV1A or 16 for 16 h (infection, blue) with serial dilutions of inocula. Newly synthesized particles were released from in parallel treated cells by three freeze/thaw cycles and inoculated on naïve HeLa cells to obtain single step growth curves (red). Infection was scored using automated image analysis. Means and SEMs of one representative triplicate are shown.

To determine optimal conditions for high content infection assays we performed time course and titration experiments with HRV1A, 2, 14, 16 and 37 and CVB3 and B4. As expected from the initial experiments (see Fig. 1B, D), the dsRNA infection assay scored a time-and dose-dependent increase of the infection index for HRV16 and CVB3 (Fig. 3A, B), and also for the other viruses (additional file 2, Fig. S2). We found that an infection at low moi (less than 0.5) for 7 h at 37°C was optimal for HRVs and CVs. Longer infection times led to cytopathic effects and loss of infected cells from the culture dish. Notably, HRV infections were similar or even more efficient at 37°C compared to at 33.5°C, whereas CVB3 and B4 infections were attenuated at 33.5°C (Fig. 3A, B, and additional file 3, Fig. S3). The strong attenuation of CVs at 33.5°C was expected. The good growth characteristics of HRVs at 37°C was consistent with recent data showing that HRVs replicate well at core body temperature [43,44] and are associated with lower respiratory tract infections [3,35,45,46]. In addition, the dsRNA mabJ2 assay detected increasing infection rates in time course experiments with all the five HRVs and both coxsackieviruses (additional file 4, Fig. S4), further confirming the specificity of the assay.

We next asked if the mabJ2 replication signal from HRV1A and 16 correlated with viral titers produced in the infected cells. We found a strong correlation between the number of infected cells detected by mabJ2 in the producer cells (dubbed 'infection') and infectious virus production by the infected cells, as determined by single step growth curves yielding more than 30-fold higher titers than inoculum (Fig. 3C). This is in close agreements with reports from the literature [47]. We conclude that mabJ2-positive cells produce infectious particles confirming that the image based dsRNA infection assay can also be used for high throughput full cycle infection assessments.

### The RNA replication assay for studies with antiviral compounds

**Figure 4:**
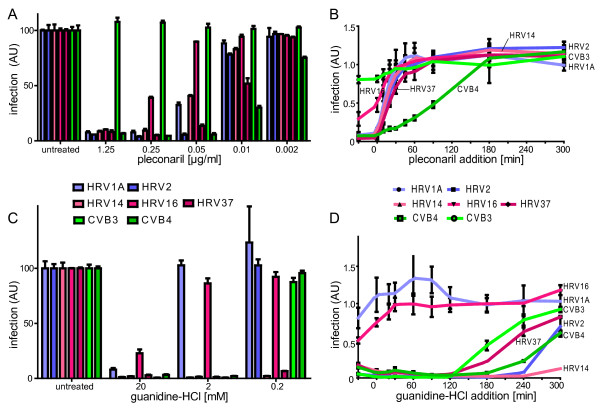
**Formation of dsRNA replication centers can be inhibited by pleconaril or guanidine-HCl**. HeLa cells were either pre-incubated with different concentrations of pleconaril (A), or pleconaril [0.5 μg/ml] was added at indicated time points before or after infection (0 min) (B). The same types of experiments were done with guanidine-HCl (C, D guanidine HCl [2 mM]). Infections with indicated HRV or CV serotypes occurred at 37°C for 7 h, and were scored by automated analysis of mabJ2. Means and SEMs of one representative triplicate are shown.

We next tested the performance of the mabJ2 dsRNA detection assay with the HRV and CV entry inhibitor pleconaril [23]. Pleconaril binds in the hydrophobic pocket of the capsid protein VP1 of several enteroviruses [48], and thereby prevents conformational changes in the capsid that enable RNA release upon receptor-mediated endocytosis. The concentration for 50% inhibition (IC50) of pleconaril in our dsRNA-based infection assay ranged from 0.01 μg/ml for the highly sensitive CVB4 up to 0.05 μg/ml to 0.1 μg/ml for the majority of HRVs (Fig. 4A, color code as in panel B). Our CVB3 strain was resistant to pleconaril in accordance with data from the literature [48].

To test if the dsRNA infection assay can be used to determine at which step of the viral life cycle a particular compound blocks infection, we performed successive compound addition experiments. Cells were treated with pleconaril either prior to infection or at defined time points post infection (pi). Pleconaril strongly inhibited infection only when added at early time points (up to about 45 min) post infection (pi) (Fig. 4B), in agreement with the notion that it inhibits the entry and conversion steps of the capsid prior to release of the RNA genome, but not genome replication [49].

To address if the dsRNA replication assay responded to downstream replication blocking agents, we treated cells with guanidine-HCl, which blocks the enteroviral protein 2C and specifically prevents the initiation of negative-strand RNA synthesis but not translation of the polyprotein [50-53]. All five HRVs (1A, 2, 14, 16, 37) and CVB3 and B4 were sensitive to the highest concentration of guanidine-HCl tested (20 mM), but HRV1A and HRV16 were not inhibited by intermediate concentrations of 2 mM (Fig. 4C), which could be related to the close genetic relationship of HRV1A and 16 [5]. The lowest concentration of guanidine (0.2 mM) inhibited HRV14 and 37, but none of the other viruses, which may also reflect the genetic diversity of the 2C protein [see for example, [5]]. Consistent with guanidine inhibition of replication but not upstream processes of infection, we found that 2 mM guanidine blocked the appearance of dsRNA mabJ2 epitopes when added up to 120 min pi for CVB3, and up to 240 min pi for the slower replicating and highly guanidine-sensitive HRV14 (Fig. 4D). The guanidine insensitive HRV1A and 16 remained rather unaffected by guanidine in the time course experiment confirming the results from the dose-dependent pre-incubation experiment (Fig. 4C). Together, these data illustrate that the dsRNA image-based replication assay is applicable for screening of small anti-viral compounds and determining the time point of their maximal efficacy in the viral replication cycle.

### Application of the RNA replication assay for image-based siRNA screens

**Figure 5:**
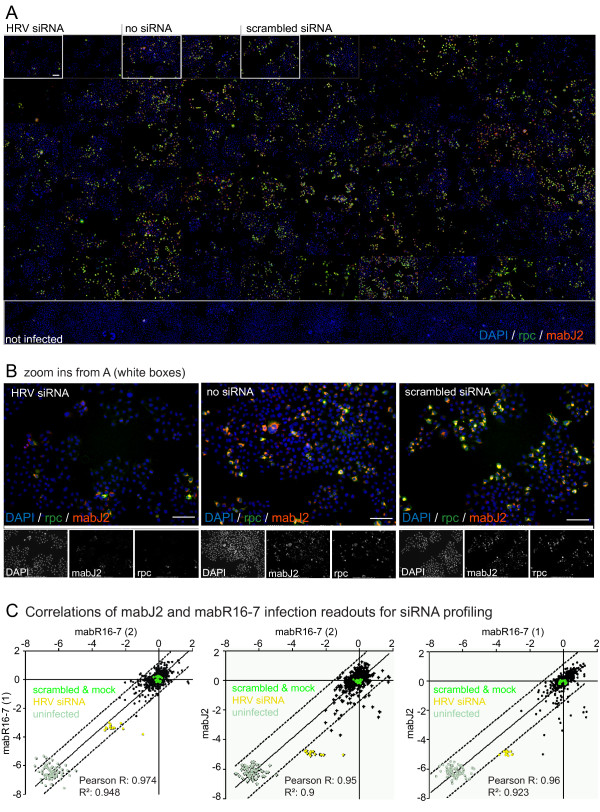
**The mabJ2 dsRNA replication assay is compatible with high content image based siRNA infection screens**. (A) Overview montage of an example siRNA screening plate. HeLa cells were infected with HRV14 and stained with a rabbit polyclonal antibody (rpc, green) raised against purified viral capsid, mabJ2 recognizing dsRNA (red) and nuclei (DAPI, blue). One out of nine images per well is shown for each siRNA, which are not specified here. (B) Examples close-ups from wells treated with HRV-targeting (HRV siRNA), no siRNA, or scrambled siRNA, followed by staining as described in (A). Merged colors are shown above, single channel micrographs are in black and white. Scale bars 100 μm. (C) Normalized HRV16 infection index (log2 transformed) determined by automated microscopy/analysis from three independent siRNA screens. Infection was measured either by mabR16-7 recognizing a VP2 epitope or mabJ2 recognizing replicated dsRNA.

siRNA profiling in cultured cells has been widely used to identify host factors with potential therapeutic impact for anti-viral or anti-microbial interference, but there were only a few genes commonly identified in the different screens. To reduce some of the technical variables for siRNA screenings in viral infections, we evaluated the mabJ2 infection assay for its applicability in high content image-based siRNA infection screens with a prototype library of 137 host factors, and a set of defined controls targeting the HRV genome, that is, three siRNA oligos per target, a total of 490 individual data points including scrambled siRNAs and-non-treated controls. Infection of HeLa cells with HRV14 was scored by mabJ2 staining and a rabbit polyclonal antibody against structural proteins of HRV14 (W.M. Lee, unpublished). Inspection of the primary imaging data revealed a strong correlation of the extent of infection determined by staining for newly synthesized viral protein or the dsRNA replication centers (Fig. 5A, B). Likewise, comparing the log2 infection indices between three independent siRNA screens of HRV16-infected HeLa cells showed strong correlations (R2 > 0.9) among the three independent replica screens using both a viral capsid specific antibody (mabR16-7) and the dsRNA infection assay (Fig. 5C). These data demonstrate that mabJ2 can be employed for detection of RNA replication centers in high throughput image-based infection screens.

### The RNA replication center assay detects infection of non-transformed human WI-38 fibroblasts

**Figure 6:**
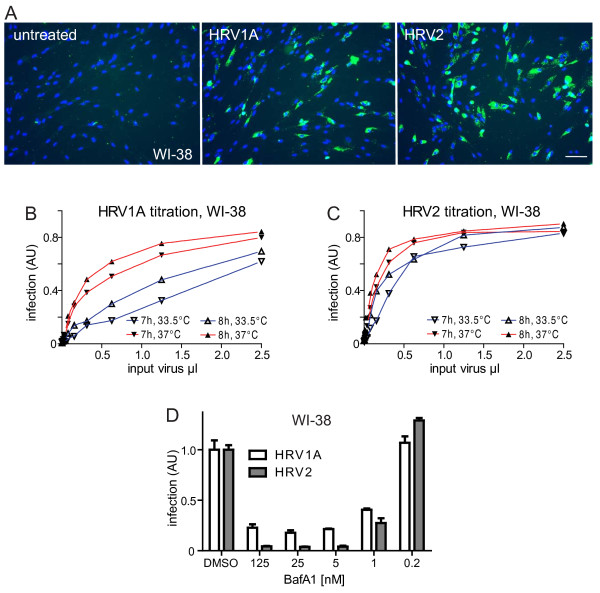
**MabJ2 detects HRV1A and 2 infections of diploid human lung airway cells**. (A) Example images of WI-38 non-transformed primary human embryonic diploid airway cells inoculated with HRV1A or HRV2 and stained for dsRNA replication centers using mabJ2 (green) and nuclei (DAPI, blue) 7 h pi. Scale bar 100 μm. (B, C) WI-38 cells were inoculated with serial dilutions of HRV1A or HRV2 for 7 or 8 h at 33.5°C (blue) or 37°C (red), and infection was quantified by the mabJ2 dsRNA infection assay using automated image acquisition/analysis. The infection index is plotted in arbitrary units (AU), where 1 means all cells infected. (D) WI-38 cells were pre-treated with increasing concentrations of bafilomycin A1 (BafA1) for 30 min, and infected with HRV1A or HRV2 for 7 h. Quantification by the mabJ2 dsRNA infection assay was by automated image acquisition/analysis and the means (n = 3) and SEMs of the normalized infection index relative to DMSO carrier control infected cells are plotted.

Finally, we also tested if mabJ2 recognized HRV-infected WI-38 primary human lung fibroblasts. We readily detected mabJ2-positive cells inoculated with the two minor group serotypes HRV1A and HRV2 (Fig. 6A). HRV1A and HRV2 infections were dependent on the temperature and inoculum dose, as indicated by analyses at 7 and 8 h pi (Fig. 6B, C). In addition, both infections were strongly attenuated by an inhibitor of the vacuolar ATPase, bafilomycin A1, in a dose-dependent manner with an IC50 of 1 nM [Fig. 6D, E, [54]]. These data were in agreement with earlier reports showing that infectious cell entry of minor group HRVs, as shown with HRV2, was dependent on low endosomal pH [55], and that both HRV1A and HRV2 were readily inactivated by low pH solutions *in vitro *[data not shown, and [56]]. To our surprise, however, the major group viruses HRV14 as well as CVB3 and B4 did not lead to detectable formation of mabJ2-positive replication centers in WI-38 cells up to 8 h pi, even at high moi (100-1000 times higher than for HeLa cells), while HRV16, HRV37 and CVA21 gave low levels of mabJ2 signals (Suppl. Fig. 5). These data show that mabJ2 detects subtle differences in infection levels in cultured cells.

## Discussion

Comprehensive studies of the vast number of enterovirus serotypes and their cell biological mechanisms of infection are a key foundation for developing new antiviral therapies. Progress in this area has been limited by the lack of reagents to detect infection of all the serotypes, and hence it has remained difficult to stringently compare the infection mechanisms from different virus serotypes or families.

Here we present a dsRNA replication center assay that can be used to detect infections by a broad range of enteroviruses in HeLa cells, that is, five human rhinovirus and three coxsackievirus serotypes. In the case of the minor HRV serotypes HRV1A and HRV2 the assay also detected infection of primary human lung WI-38 fibroblasts. The assay is applicable for high content screening, and infection readouts are time, dose and temperature-dependent.

Importantly, our assay is compatible with siRNA screening approaches, which have received considerable attention in the last few years, due to the promise to uncover much of the so far hidden host functions that support viral infections. Recently genome wide or subgenomic screens have been published for a variety of viral pathogens, including HIV [57-59], HCV [60,61], dengue virus [62], West Nile virus [63], influenza virus [64-68], human papillomavirus [69] and vaccinia virus [70]. The multiple screens for HIV, influenza virus and HCV, however, identified only very few overlapping genes for the individual viruses. Reasons for such findings have been attributed to the biological nature of cells and viruses, including virus strain differences, cell line differences, cell context-dependent effects and redundancies of host factors. Among the technical reasons for the low levels of overlapping hits from the published screens are also the different sources and efficacies of siRNAs, which depended on the manufacturer, or whether single siRNAs or siRNA pools were used. In addition, the different hit scoring algorithms, including post-processing filters and variable accounts for toxicity and specificity, hit ranking algorithms, or consideration of hit assignment to previously known functional networks of cellular pathways can contribute to different hit lists from siRNA screens. Last but not least, the assays for infection are not standardized, that is, different types of infection assays cover variable phases of the viral replication cycle with variable efficacies and, hence, detection sensitivities and hit identifications are poorly informed.

Our data support the notion that mabJ2 detects replicating dsRNA in infected cells rather than genomic RNA from incoming virus particles. MabJ2 is hence useful to measure viral replication. We suggest that mabJ2 (or any similar antibody) can be used to detect infections of any positive-strand RNA virus that is actively replicating. It may even be used to detect dsRNA from certain DNA virus infections [71]. These findings and the fact that mabJ2 detects dsRNA with high sensitivity in solid support based assays [31] open a path towards standardized and reproducible infection assays, and possibly clinical diagnostics.

Our dsRNA replication assay was validated at several levels. The dsRNA readout correlated with single step growth curves, whereby the infectious titers produced per cell were similar to values reported in the literature, that is, in the range of 40 plaque forming units per cell [47]. We have also validated the assay with two proof of concept chemical compounds known to block enterovirus infections, the capsid binding component pleconaril [23,72] and the 2C protein inhibitor guanidine [50]. While pleconaril was an entry inhibitor with a half maximal inhibition time of about 25 to 30 min, guanidine blocked infection until 2 to 4 h pi, reflecting the different modes of action of these compounds. Hence, our dsRNA replication assay in the image-based high content format may prove useful also for screening of small chemical libraries against viral infections.

## Conclusions

The mabJ2 RNA replication assay has proven to be a reliable procedure to study enterovirus infections on a systematic level opening new doors for comparative genomic and chemical studies. It fulfils requirements such as robustness, good signal-to-noise ratio and practical usability, making it broadly and systematically applicable for high content infection assays for enteroviruses, and possibly other plus-sense RNA viruses. The assay covers steps required for virus entry, translation and RNA replication, and can be extended to a full replication cycle assay. It is based on a commercially available mouse monoclonal antibody, which is readily accessible for both academic and commercial laboratories. The assay also offers a way to carry out mechanistic studies with many different serotypes, including emerging picornaviruses, and hence identify serotype independent requirements for picornavirus infection.

## Methods

### Cell culture and virus production

HeLa cervical carcinoma cells strain Ohio (from L. Kaiser; Central Laboratory of Virology, University Hospital Geneva, Switzerland) and primary human embryonic lung WI-38 cells [American Type Culture Collection, [73]] were cultured in Dulbecco's Modified Eagle Medium (Sigma-Aldrich) supplemented with L-glutamine (Sigma-Aldrich), non-essential amino acids (Sigma-Aldrich) and 10% fetal calf serum (FCS, Sigma-Aldrich) at 37°C and 5% CO_2 _in a humidified incubator. In all experiments passage numbers were kept at a maximum of 25 post thawing. For infection experiments in 96 well imaging plates (Matrix) 14,000 cells were split in a total of 100 μl the day before the experiment. HRV serotypes 1A and16 were provided by W.M. Lee (Department of Pediatrics, School of Medicine and Public Health, University of Wisconsin, Madison, Wisconsin, USA), HRV2, 14 and 37 were from L. Kaiser and CVB3, B4 and A21 were from T. Hyypiä (Department of Virology, University of Turku, Finland).

Both HRVs and CVs were grown in HeLa cells. Briefly, cells were inoculated with a cell lysate stock from the respective serotypes at 33.5°C (HRV) or 37°C (CV) over night in infection media (IM/FC-DMEM supplemented with L-glutamine, 30 mM MgCl_2 _and 2% FCS). When CPE was visible in 80-90% of the cells, media was removed and cells harvested by scraping and pelleting, lysed by 3 freeze/thaw cycles and centrifuged at 2500 × g for 10 min. Aliquots of the supernatants containing stock virus were stored at -80°C. All serotypes used in this study were analyzed by reverse transcriptase-polymerase chain reaction and diagnostic sequencing of the 5'UTR and/or capsid regions and found to be virtually identical with the published sequences. For details, see additional files 5, 6, 7, 8.

### Infections and immunocytochemistry

Viruses where added to cells in infection media/BSA (DMEM supplemented with L-glutamine, 30 mM MgCl_2 _and 0.2% BSA, Sigma-Aldrich). For all the compound and siRNA experiments, moi was chosen such that approximately 20 to 40% of the cells were infected at 7 h pi. Cells were fixed by adding 1/3 volume of 16% para-formaldehyde directly to the cells in culture media. Fixation was for either 15 min at room temperature or long term storage at 4°C. Cells were washed with PBS, PBS/25 mM NH_4_Cl and PBS, permeabilized with 0.2% Triton X-100 (Sigma-Aldrich) and washed twice with PBS and blocked with PBS containing 1% BSA (Fraction V, Sigma). Antibodies detecting viral protein antigens were used as follows: for HRV1A and HRV16 mabR16-7 [35], for HRV2 mab8F5 [74], for HRV14, 37 and CVB3, B4 the rabbit polyclonal antisera (rpc, W.M. Lee, unpublished). MabJ2 and K1 used to detect dsRNA of infected cells [31,71] were obtained from English & Scientific Consulting (Bt. Szirák, Hungary). Fixed and permeabilized cells were incubated at room temperature for 1 h with diluted mabJ2 in PBS/1%BSA (0.33 μg/ml which corresponded to a 1:1500 dilution of the 0.5 mg/ml antibody). Cells were washed twice with PBS and incubated with Alexa-fluor labelled secondary antibodies (Invitrogen) at 0.2 μg/ml for 1 h. Nuclei were stained with DAPI, and cells on coverslips mounted in mounting media (Dako), or the 96 well imaging plates were stored at 4°C in PBS/NaN_3_.

### Automated image acquisition and data analysis

Automated image acquisition was performed with an ImageXpress Micro (Molecular Devices) equipped with a CoolSNAP HQ 12bit greyscale camera (Roper Scientific) and 10×/NA 0.5 objective (Nikon). Routinely, 9-20 images per 96 well were acquired leading to an average of 5000-12000 cells analyzed per well. For high resolution images, an Olympus IX81 equipped with a 60×/1.4 NA. objective and oil immersion was used. Image overlays were made using MetaXpress (Molecular Devices) and ImageJ (NIH Image, http://rsbweb.nih.gov/nih-image/). Images were analyzed using a custom written Matlab routine. Briefly, a canny edge algorithm was used to identify areas of all the nuclei stained with DAPI [75] and infected cells stained for newly synthesized viral protein or replicating dsRNA were identified by a user-defined thresholding method scoring staining intensity and size. If the overlap of the nuclear and infection signals exceeded a user defined threshold, a cell was scored as infected. Data analysis was performed using Prism (version 5.01, Graphpad), and data for different serotypes were plotted in the order of HRV1A, 2, 14, 16, 37, and CVB3, B4 as infection indices (fraction of infected cells per total cell number, indicated as arbitrary units) unless stated otherwise.

### ICAM-1 receptor blocking and compound assays

HeLa cells were pre-incubated with mouse monoclonal anti-ICAM-1 antibody mab15.2L (Santa Cruz) at 37°C at a concentration of 0.5 μg of antibody in 50 μl of infection medium/BSA per 96 well for 1 h, followed by infection for 7 h and staining for dsRNA replication centers. For compound assays cells were pre-incubated for 30 min with compounds diluted in infection medium/BSA prior to virus addition. Virus diluted in infection medium/BSA was added to the cells at 37°C for 7 h, and cells were fixed and immunostained. All compounds were dissolved in dimethyl sulfoxid (DMSO, cell culture grade, Sigma-Aldrich) and the respective concentrations of DMSO were used as controls. Pleconaril was a kind gift from 3-V Biosciences and guanidine-HCl was bought from Sigma-Aldrich.

### siRNA screens

For siRNA experiments, siRNA oligos (Qiagen) were spotted in OptiMEM-I (Gibco) at a final concentration of 50 nM in 96 well imaging plates (Matrix). Lipofectamine 2000 (Invitrogen)/OptiMEM-I was added to a total volume of 25 μl, and 3000 HeLa cells were seeded into each 96 well in a total of 100 μl per well. Transfected cells were incubated for 65 h, followed by infection at 37°C for 7 h and fixation/staining as indicated above. Specific siRNA oligos directed against the structural protein VP4 (termed HRV siRNA) were designed according to the specific genomic sequence of the particular serotype [76].

## Competing interests

The project was in part financially supported by a grant from 3-V Biosciences Inc (Zurich, Switzerland, and Menlo Park, CA, USA), the Swiss National Science Foundation, the Swiss SystemsX.ch initiative, grant InfectX and the Kanton Zurich to UFG. The funders had no role in study design, data collection and analysis or preparation of the manuscript. UFG is a founder of 3-V Biosciences, and UFG and SM are shareholders of 3-V Biosciences.

## Authors' contributions

AJ set up and optimized the assay and performed all experiments documented by figures; UFG had the initial idea to test mabJ2 in high content infection screening; SM provided the Matlab code for analysis of infection experiments; AD, PR, AJ and ML designed and performed the diagnostic sequencing of HRVs and CVs; WML provided essential antibodies and protocols for virus growth, UFG & AJ wrote the manuscript.All authors have read and approved the final manuscript.

## Supplementary Material

Additional file 1 **Fig. S1**. Automated image analysis details. The matlab scoring algorithm (1) detects edges of the nuclei (A, DAPI) and infection (B, immunostaining) channels using a canny edge algorithm and user defined thresholds and forms areas by closing the edges. (2) Areas below or above a set size-threshold are excluded from both channels (A2, B2) leading to the final total cell (A3) and infection (B3) mask. Merging of both masks leads to the final result indicating infected and not infected cells (as shown in Fig. 1C). Scale bar corresponds to 100 μm. Click here for file

Additional file 2 **Fig. S2**. Dose and temperature dependent formation of dsRNA replication centers of HRV1A, 2, 14, 37 or CVB4 infected HeLa cells. The dose dependencies of HRV1A, 2, 14, 37 and CVB4 infections at 33.5° (blue) or 37°C (red) were determined for the mabJ2 dsRNA infection assay in HeLa cells by two fold serial dilutions of inocula. Infection was scored using automated image acquisition/analysis. Means and SEMs of one representative triplicate are shown. Click here for file

Additional file 3 **Fig. S3**. Time and temperature dependent formation of dsRNA replication centers of HRV1A, 2, 14, 37 and CVB4 and A21 infected HeLa cells. The time dependencies of of HRV1A, 2, 14, 37 and CVB4 and A21 infections at 33.5°C (blue) or 37°C (red) were determined for the mabJ2 dsRNA infection assay in HeLa cells by infection for 300 to 700 min. Infections were scored using automated image analysis. Means and SEMs of one representative triplicate are shown. Click here for file

Additional file 4 **Fig. S4**. MabJ2 dsRNA replication center assay in normal human lung airway cells. (A) Example images of WI-38 non-transformed primary human embryonic diploid airway cells inoculated with the indicated HRV and CV serotypes and stained for dsRNA replication centers using mabJ2 (green) and nuclei (DAPI, blue) 7 h pi. Scale bar 100 μm. (B) WI-38 cells were inoculated with serial dilutions of the indicated HRV and CV serotypes for 7 or 8 h at 33.5°C (blue) or 37°C (red), and infection was quantified by the mabJ2 dsRNA infection assay using automated image acquisition/analysis. The infection index is plotted in arbitrary units (AU), where 1 means all cells infected. Click here for file

Additional file 5 **Table S1**. List of primers for diagnostic sequencing of HRV and CV serotypes Click here for file

Additional file 6 **Table S2**. Top results of Blastn alignments of HRV and CV diagnostic PCR products Click here for file

Additional file 7 **Table S3**. DNA sequences of reverse transcribed PCR products from five HRV and two CV serotypes Click here for file

Additional file 8 **Supplemental references**. Click here for file
