# Supplementary material for: An RNA replication-center assay for high content image-based quantifications of human rhinovirus and coxsackievirus infections
Source: Virol J. 2010 Oct 11;7:264. doi: 10.1186/1743-422X-7-264 (PMC2958916; doi:10.1186/1743-422X-7-264)
Supplement: Additional file 1 — Fig. S1. Automated image analysis details. The matlab scoring algorithm (1) detects edges of the nuclei (A, DAPI) and infection (B, immunostaining) channels using a canny edge algorithm and user defined thresholds and forms areas by closing the edges. (2) Areas below or above a set size-threshold are excluded from both channels (A2, B2) leading to the final total cell (A3) and infection (B3) mask. Merging of both masks leads to the final result indicating infected and not infected cells (as shown in Fig. 1C). Scale bar corresponds to 100 μm. [file 1743-422X-7-264-S1.PDF]

Fig. S1

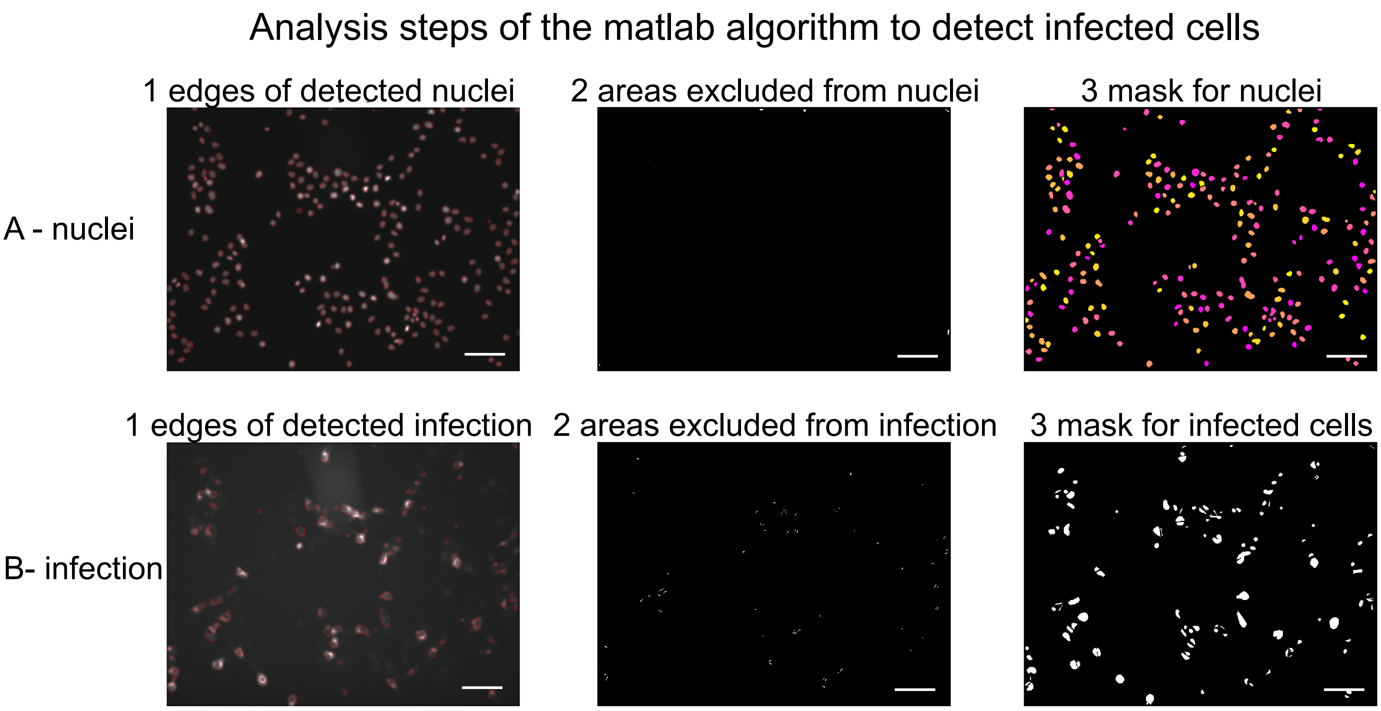

Additional file 1, Fig. S1: Automated image analysis.

The matlab scoring algorithm (1) detects edges of the nuclei (A, DAPI) and infection (B, immunostaining) channels using a canny edge algorithm and user defined thresholds and forms areas by closing the edges. (2) Areas below or above a set size-threshold are excluded from both channels (A2, B2) leading to the final total cell (A3) and infection (B3) mask. Merging of both masks leads to the final result indicating infected and not infected cells (as shown in Fig. 1C). Scale bar corresponds to 100 μm.
