# Supplementary material for: An RNA replication-center assay for high content image-based quantifications of human rhinovirus and coxsackievirus infections
Source: Virol J. 2010 Oct 11;7:264. doi: 10.1186/1743-422X-7-264 (PMC2958916; doi:10.1186/1743-422X-7-264)
Supplement: Additional file 2 — Fig. S2. Dose and temperature dependent formation of dsRNA replication centers of HRV1A, 2, 14, 37 or CVB4 infected HeLa cells. The dose dependencies of HRV1A, 2, 14, 37 and CVB4 infections at 33.5° (blue) or 37°C (red) were determined for the mabJ2 dsRNA infection assay in HeLa cells by two fold serial dilutions of inocula. Infection was scored using automated image acquisition/analysis. Means and SEMs of one representative triplicate are shown. [file 1743-422X-7-264-S2.PDF]

Fig. S2

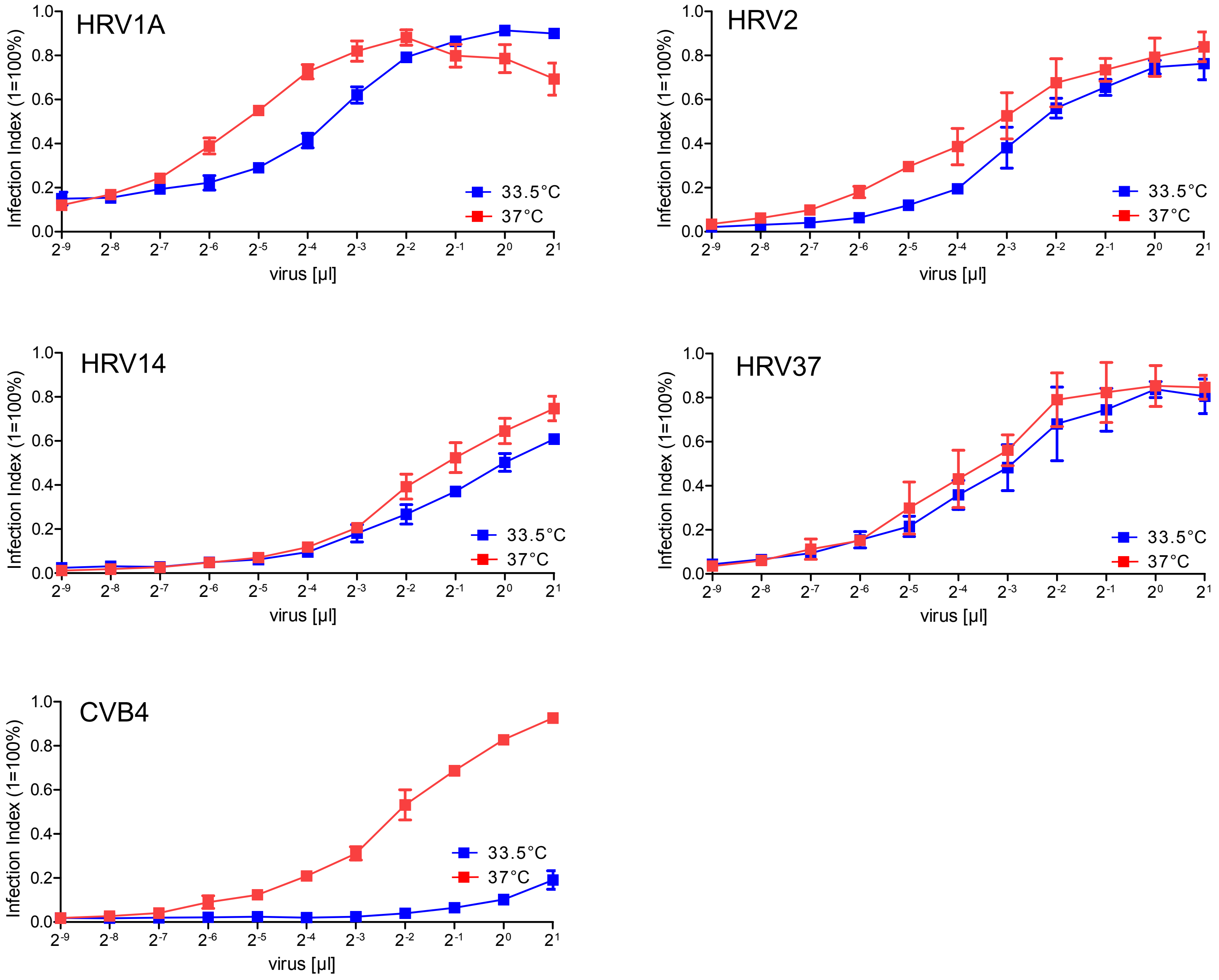

Additional file 2, Fig. S2: Dose and temperature dependent formation of dsRNA replication centers of HRV1A, 2, 14, 37 or CVB4 infected HeLa cells.

The dose dependencies of HRV1A, 2, 14, 37 and CVB4 infections at 33.5° (blue) or 37°C (red) were determined for the mabJ2 dsRNA infection assay in HeLa cells by two fold serial dilutions of inocula. Infection was scored using automated image acquisition/analysis. Means and SEMs of one representative triplicate are shown.
