# Supplementary material for: An RNA replication-center assay for high content image-based quantifications of human rhinovirus and coxsackievirus infections
Source: Virol J. 2010 Oct 11;7:264. doi: 10.1186/1743-422X-7-264 (PMC2958916; doi:10.1186/1743-422X-7-264)
Supplement: Additional file 3 — Fig. S3. Time and temperature dependent formation of dsRNA replication centers of HRV1A, 2, 14, 37 and CVB4 and A21 infected HeLa cells. The time dependencies of of HRV1A, 2, 14, 37 and CVB4 and A21 infections at 33.5°C (blue) or 37°C (red) were determined for the mabJ2 dsRNA infection assay in HeLa cells by infection for 300 to 700 min. Infections were scored using automated image analysis. Means and SEMs of one representative triplicate are shown. [file 1743-422X-7-264-S3.PDF]

Fig. S3

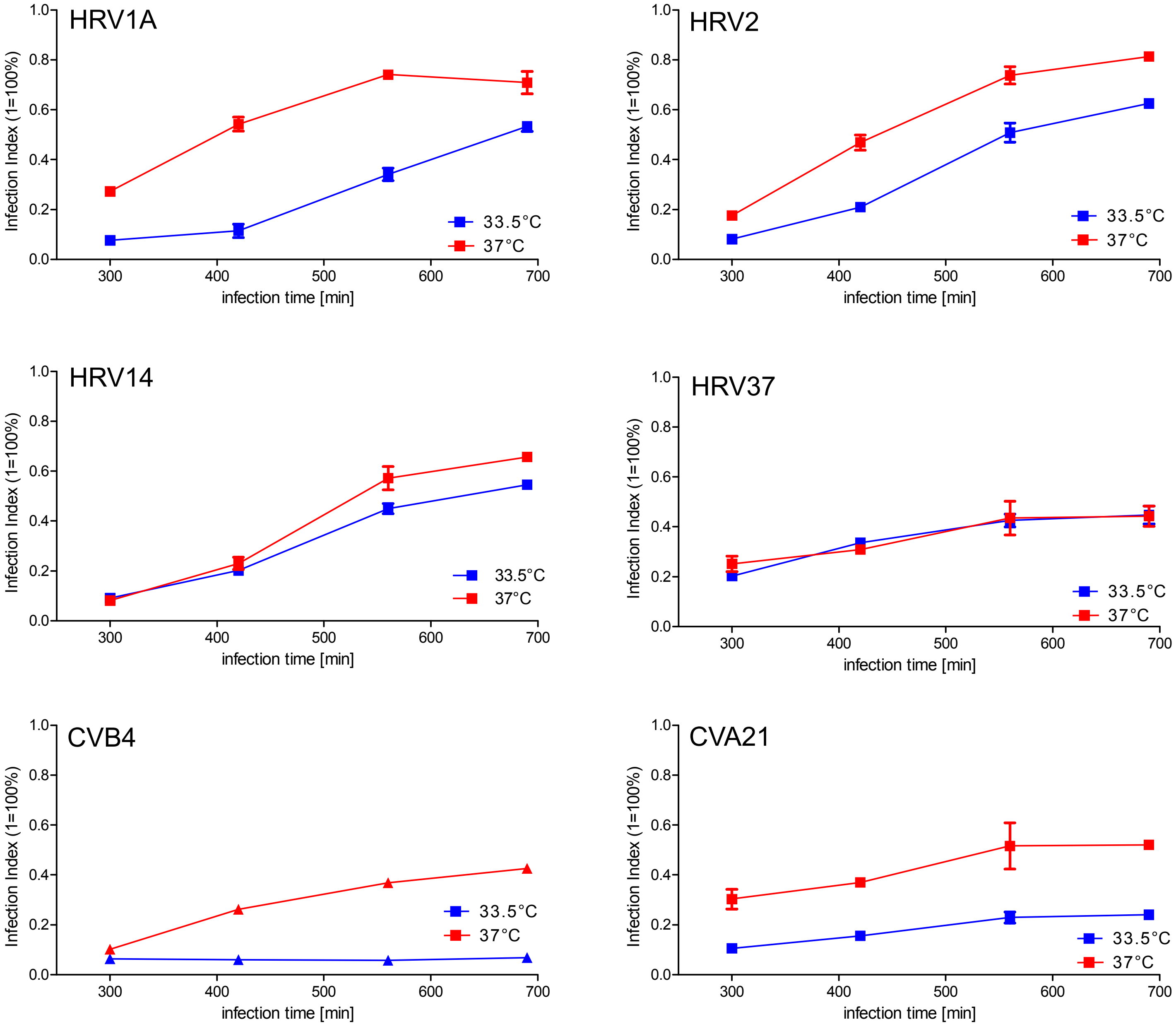

Additional file 3, Fig. S3: Time and temperature dependent formation of dsRNA replication centers of HRV1A, 2, 14, 37 and CVB4 and A21 infected HeLa cells.

The time dependencies of of HRV1A, 2, 14, 37 and CVB4 and A21 infections at 33.5°C (blue) or 37°C (red) were determined for the mabJ2 dsRNA infection assay in HeLa cells by infection for 300 to 700 min. Infections were scored using automated image analysis.
Means and SEMs of one representative triplicate are shown.
