# Supplementary material for: An RNA replication-center assay for high content image-based quantifications of human rhinovirus and coxsackievirus infections
Source: Virol J. 2010 Oct 11;7:264. doi: 10.1186/1743-422X-7-264 (PMC2958916; doi:10.1186/1743-422X-7-264)
Supplement: Additional file 4 — Fig. S4. MabJ2 dsRNA replication center assay in normal human lung airway cells. (A) Example images of WI-38 non-transformed primary human embryonic diploid airway cells inoculated with the indicated HRV and CV serotypes and stained for dsRNA replication centers using mabJ2 (green) and nuclei (DAPI, blue) 7 h pi. Scale bar 100 μm. (B) WI-38 cells were inoculated with serial dilutions of the indicated HRV and CV serotypes for 7 or 8 h at 33.5°C (blue) or 37°C (red), and infection was quantified by the mabJ2 dsRNA infection assay using automated image acquisition/analysis. The infection index is plotted in arbitrary units (AU), where 1 means all cells infected. [file 1743-422X-7-264-S4.PDF]

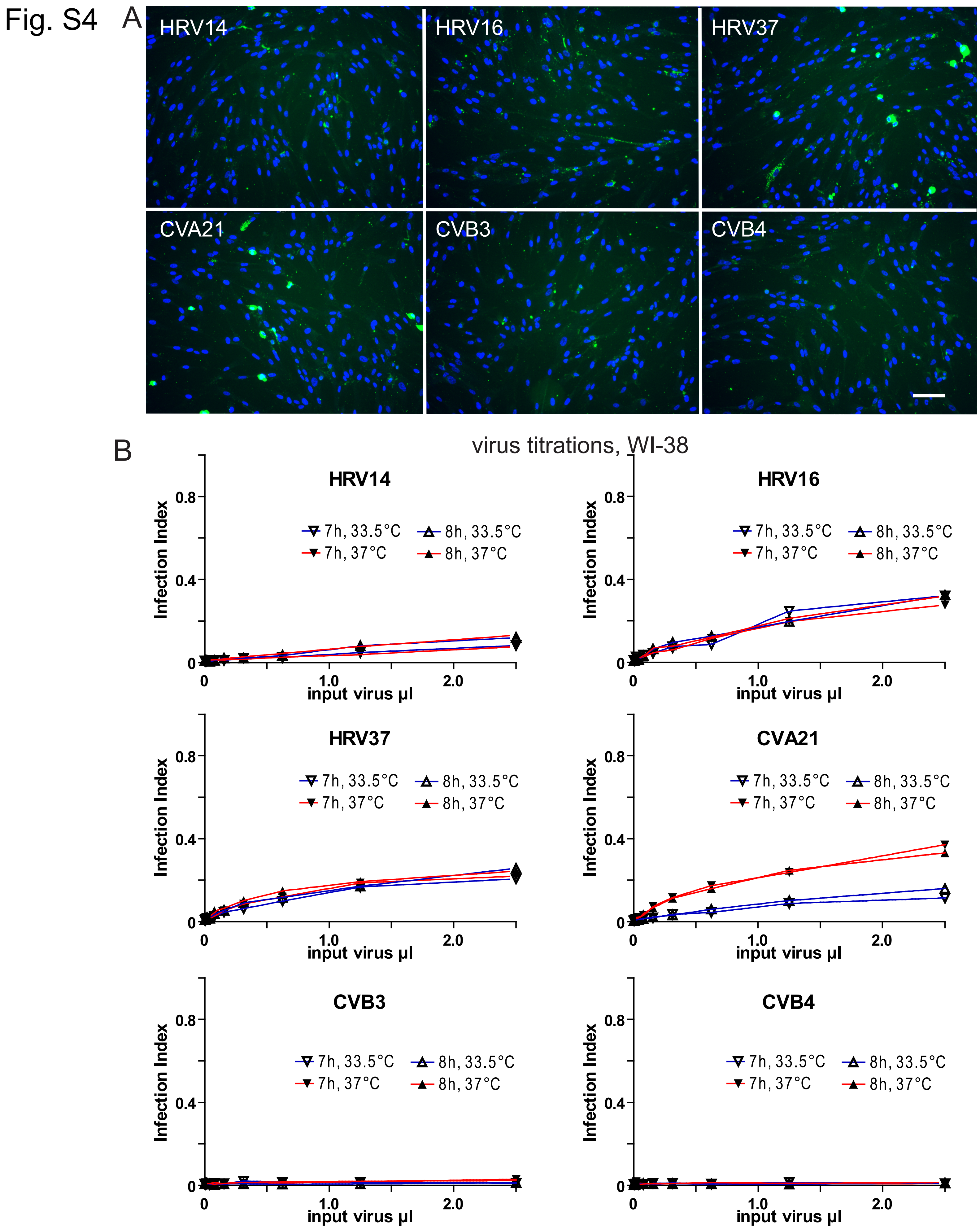

Additional file 4, Fig. S4: MabJ2 dsRNA replication center assay in normal human lung airway cells.

(A) Example images of WI-38 non-transformed primary human embryonic diploid airway cells inoculated with the indicated HRV and CV serotypes and stained for dsRNA replication centers using mabJ2 (green) and nuclei (DAPI, blue) 7 h pi. Scale bar 100 µm.
(B) WI-38 cells were inoculated with serial dilutions of the indicated HRV and CV serotypes for 7 or 8 h at 33.5°C (blue) or 37°C (red), and infection was quantified by the mabJ2 dsRNA infection assay using automated image acquisition/analysis.
The infection index is plotted in arbitrary units (AU), where 1 means all cells infected.
