# Supplementary material for: An RNA replication-center assay for high content image-based quantifications of human rhinovirus and coxsackievirus infections
Source: Virol J. 2010 Oct 11;7:264. doi: 10.1186/1743-422X-7-264 (PMC2958916; doi:10.1186/1743-422X-7-264)
Supplement: Additional file 5 — Table S1. List of primers for diagnostic sequencing of HRV and CV serotypes [file 1743-422X-7-264-S5.DOC]

**Additional file 5, Table S1: List of primers for diagnostic sequencing of HRV and CV serotypes**

| **primer** | **sense** | **sequence (5’ –> 3’)** |
| --- | --- | --- |
| WML-FWD | fwd | CAAGCACTTCTGTTTCCCC |
| Primer 1-REV | rev | GAAACACGGACACCCAAAGTA |
| WML-1AB-REV | rev | TCAACAGATGGTGATTGTAGTG |
| WML-2-REV | rev | CCTATCAGAGTATCCACAAGC |
| RV14gustin-REV | rev | TGATTGACCAGCTGATGATG |
| WML-16-REV | rev | TAGTTTCCACCACCAACC |
| WML-37-REV | rev | ATCTACATACAGAAGTGTCTGG |
| CVB3-VP12A-FWD | fwd | ATGACGGATGGTCTGAATTTTCCAGGAACG |
| CVB3-VP12A-REV | rev | CTCTTGGACCTCTACTAGACCTGGTCCTTC |
| CVB4-VP12A-FWD | fwd | ACCATGTTTTATGACGGGTGGTCAAACTTC |
| CVB4-VP12A-REV | rev | ACTGGTATCTTTTTGGGTAATATTCACTTT |
| CVA21-VP12A-FWD | fwd | ACGTAGGGATTGCCAATGCCTATTCACACT |
| CVA21-VP12A-REV | rev | TATCTTGCTGGATAGTAGTCGTTTGCTTCC |

All the serotypes used in this study were diagnostically analyzed by RT-PCR (reverse transcriptase-polymerase chain reaction) and sequencing using previously established methods [1-4]. Total RNA from infected HeLa cells was extracted using TrIzol reagent (Sigma-Aldrich), and cDNAs were obtained by reverse transcription with the SuperScript III kit (Invitrogen) according to manufacturer’s instructions. Fragments were amplified by PCR with the Taq DNA polymerase (Sigma-Aldrich) and sets of primers (listed below). The 5’ UTR region of all HRV serotypes was amplified using the primers WML-FWD and primer 1-REV, and the 5’ UTR/VP2 region using the forward primer WML-FWD and the specific reverse primers for the different HRV serotypes WML-1AB-REV (HRV1A), WML-2 REV (HRV2), RV14gustin-REV (HRV14), WML-16-REV (HRV16) and WML-37 REV (HRV37). The VP1/2A region of all CV serotypes was amplified using the primers CVB3-VP12A-FWD and CVB3-VP12A-REV (CVB3), CVB4-VP12A-FWD and CVB4-VP12A-REV (CVB4), and CVA21-VP12A-fwd and CVA21-VP12A-REV (CVA21). The amplified fragments were purified, sequenced and blasted against a pool of known human genome and transcript nucleotide sequences (<http://blast.ncbi.nlm.nih.gov/Blast.cgi>) (see additional file 6, Table S2).
