# Supplementary material for: An RNA replication-center assay for high content image-based quantifications of human rhinovirus and coxsackievirus infections
Source: Virol J. 2010 Oct 11;7:264. doi: 10.1186/1743-422X-7-264 (PMC2958916; doi:10.1186/1743-422X-7-264)
Supplement: Additional file 6 — Table S2. Top results of Blastn alignments of HRV and CV diagnostic PCR products [file 1743-422X-7-264-S6.DOC]

**Additional file 6, Table S2: Top results of Blastn alignments of HRV and CV diagnostic PCR products**

| **Serotype** | **Fragment type** | **Accession** | **Description** | **E-value** |
| --- | --- | --- | --- | --- |
| **HRV1A** | 5’ UTR | AF108179.1 | Human rhinovirus serotype 1A 5’ untranslated region | 3e-169 |
| **HRV1A** | 5’ UTR/VP2 | FJ445111.1 | Human rhinovirus 1 strain ATCC VR-1559, complete genome | 0.0 |
| **HRV2** | 5’ UTR | EU095989.1 | Human rhinovirus 2 5’ UTR | 2e-166 |
| **HRV2** | 5’ UTR/VP2 | X02316.1 | Human rhinovirus 2, complete sequence | 0.0 |
| **HRV14** | 5’ UTR | EU096001.1 | Human rhinovirus 14 5’ UTR | 1e-173 |
| **HRV14** | 5’ UTR/VP2 | K02121.1 | Human rhinovirus type 14 (HRV14), complete genome | 0.0 |
| **HRV16** | 5’ UTR | EU096003.1 | Human rhinovirus 16 5’ UTR | 1e-172 |
| **HRV16** | 5’ UTR/VP2 | L24917.1 | Human rhinovirus type 16 polyprotein gene, complete CDA | 0.0 |
| **HRV37** | 5’ UTR | EU096024.1 | Human rhinovirus 37 5’ UTR | 1e-173 |
| **HRV37** | long | EF173423.1 | Human rhinovirus 37, complete genome | 0.0 |
| **CVB3** | VP1/2A | AY752944 | Human coxsackievirus B3 strain 28, complete genome | 0.0 |
| **CVB4** | VP1/2A | X05690 | Coxsackievirus B4 complete genome | 0.0 |
| **CVA21** | VP1/2A | AF546702 | Human coxsackievirus A21 strain Kuykendall, complete genome | 0.0 |
