# Supplementary material for: An RNA replication-center assay for high content image-based quantifications of human rhinovirus and coxsackievirus infections
Source: Virol J. 2010 Oct 11;7:264. doi: 10.1186/1743-422X-7-264 (PMC2958916; doi:10.1186/1743-422X-7-264)
Supplement: Additional file 7 — Table S3. DNA sequences of reverse transcribed PCR products from five HRV and two CV serotypes [file 1743-422X-7-264-S7.DOC]

**Additional file 7, Table S3: DNA sequences of reverse transcribed PCR products from five HRV and two CV serotypes**

Two primer pairs for the indicated viruses designated (a) and (b), were used. For details, see additional file 5, Table S1.

Sequences are accessible at GenBank, accession numbers:

BankIt1396055 Seq1 HQ336413 HRV1a 5UTR-VP2

BankIt1396055 Seq2 HQ336414 HRV2 5UTR-VP2

BankIt1396055 Seq3 HQ336415 HRV14 5UTR-VP2

BankIt1396055 Seq4 HQ336416 HRV16 5UTR-VP2

BankIt1396055 Seq5 HQ336417 HRV37 5UTR-VP2

BankIt1396055 Seq6 HQ336418 CVA21 VP1-2A

BankIt1396055 Seq7 HQ336419 CVB3 VP1-2A

BankIt1396055 Seq8 HQ336420 CVB4 VP1-2A

**HRV1a_5UTR.ab1_(a)**

GATACCGTTATCCGCAAGTGCCTACACAGAGCTTAGTAGGATTCTGAAAGATCTTTGGTTGGTCGTTCAGCTGCATACCCAGCAGTAGACCTTGCAGATGAGGCTGGACATTCCCCACTGGTAACAGTGGTCCAGCCTGCGTGGCTGCCTGCGCACCTCTCATGAGGTGTGAAGCCAAAGATCGGACAGGGTGTGAAGAGCCGCGTGTGCTCACTTTGAGTCCTCCGGCCCCTGAATGCGGCTAACCTTAAACCTGCAGCCATGGCTCATAAGCCAATGAGTTTATGGTCGTAATGAGTAATTGCGGGATGGGACCGACTACTTTGGGTGTCCGTGTTTCA

**HRV1a_5UTRVP2.ab1_(b)**

CACAGATACCGTTATCCGCAAGTGCCTACACAGAGCTTAGTAGGATTCTGAAAGATCTTTGGTTGGTCGTTCAGCTGCATACCCAGCAGTAGACCTTGCAGATGAGGCTGGACATTCCCCACTGGTAACAGTGGTCCAGCCTGCGTGGCTGCCTGCGCACCTCTCATGAGGTGTGAAGCCAAAGATCGGACAGGGTGTGAAGAGCCGCGTGTGCTCACTTTGAGTCCTCCGGCCCCTGAATGCGGCTAACCTTAAACCTGCAGCCATGGCTCATAAGCCAATGAGTTTATGGTCGTAATGAGTAATTGCGGGATGGGACCGACTACTTTGGGTGTCCGTGTTTCACTTTTTCCTTTATTAATTGCTTATGGTGACAATATATATATTGATATATATTGGCATCATGGGCGCCCAGGTATCTAGACAAAATGTTGGTACACACTCAACCCAAAATTCAGTGTCAAATGGATCAAGTTTAAATTACTTTAATATAAATTACTTCAAGGATGCTGCCTCAAGTGGTGCATCTAGATTAGATTTCTCTCAAGATCCAAGCAAATTCACTGACCCAGTTAAAGATGTCTTAGAAAAGGGGATCCCAACACTACAATCAC

**HRV2_5UTR.ab1_(a)**

ACTGCGATCGTTATCCGCAAGCGCCTACGCAAAGCTTAGTAGCATCTTTGAAATCGTTTGGCTGGTCGATCCGCCATTTCCCCTGGTAGACCTGGCAGATGAGGCTAGAAATACCCCACTGGCGACAGTGTTCTAGCCTGCGTGGCTGCCTGCACACCCTATGGGTGTGAAGCCAAACAATGGACAAGGTGTGAAGAGCCCCGTGTGCTCGCTTTGAGTCCTCCGGCCCCTGAATGTGGCTAACCTTAACCCTGCAGCTAGAGCACGTAACCCAACGTGTATCTAGTCGTAATGAGCAATTGCGGGATGGGACCAACTACTTTGGGTGTCCGTGTTT

**HRV2_5UTRVP2.ab1_(b)**

ACTGCGATCGTTATCCGCAAGCGCCTACGCAAAGCTTAGTAGCATCTTTGAAATCGTTTGGCTGGTCGATCCGCCATTTCCCCTGGTAGACCTGGCAGATGAGGCTAGAAATACCCCACTGGCGACAGTGTTCTAGCCTGCGTGGCTGCCTGCACACCCTATGGGTGTGAAGCCAAACAATGGACAAGGTGTGAAGAGCCCCGTGTGCTCGCTTTGAGTCCTCCGGCCCCTGAATGTGGCTAACCTTAACCCTGCAGCTAGAGCACGTAACCCAACGTGTATCTAGTCGTAATGAGCAATTGCGGGATGGGACCAACTACTTTGGGTGTCCGTGTTTCACTTTTTCCTTTATATTTGCTTATGGTGACAATATATACAATATATATATTGGCACCATGGGTGCACAGGTTTCAAGACAAAATGTTGGAACTCACTCCACGCAAAACTCTGTATCAAATGGGTCTAGTTTAAATTATTTTAACATCAATTATTTCAAAGATGCTGCTTCAAATGGTGCATCAAAACTGGAATTCACACAAGATCCTAGTAAATTTACTGACCCAGTTAAGGATGTTTTGGAAAAGGGAATACCAACACTACAGTCCCCCACAGTGGAGGCTTGTG

**HRV14_5UTR.ab1_(a)**

CCTTTACCGTTATCCGCCAACCAACTACGTAACAGTTAGTACCATCTTGTTCTTGACTGGACGTTCGATCAGGTGGATTTTCCCTCCACTAGTTTGGTCGATGAGGCTAGGAATTCCCCACGGGTGACCGTGTCCTAGCCTGCGTGGCGGCCAACCCAGCTTATGCTGGGACGCCCTTTTAAGGACATGGTGTGAAGACTCGCATGTGCTTGGTTGTGAGTCCTCCGGCCCCTGAATGCGGCTAACCTTAACCCTGGAGCCTTATGCCACGATCCAGTGGTTGTAAGGTCGTAATGAGCAATTCCGGGACGGGACCGACTACTTTGGGTGTCCGTGTTTC

**HRV14_5UTRVP2.ab1_(b)**

CCTTTACCGTTATCCGCCAACCAACTACGTAACAGTTAGTACCATCTTGTTCTTGACTGGACGTTCGATCAGGTGGATTTTCCCTCCACTAGTTTGGTCGATGAGGCTAGGAATTCCCCACGGGTGACCGTGTCCTAGCCTGCGTGGCGGCCAACCCAGCTTATGCTGGGACGCCCTTTTAAGGACATGGTGTGAAGACTCGCATGTGCTTGGTTGTGAGTCCTCCGGCCCCTGAATGCGGCTAACCTTAACCCTGGAGCCTTATGCCACGATCCAGTGGTTGTAAGGTCGTAATGAGCAATTCCGGGACGGGACCGACTACTTTGGGTGTCCGTGTTTCTCATTTTTCTTCATATTGTCTTATGGTCACAGCATATATATACATATACTGTGATCATGGGCGCTCAGGTTTCTACACAGAAAAGTGGATCTCACGAAAATCAAAACATTTTGACCAATGGATCAAATCAGACTTTCACAGTTATAAATTACTATAAGGATGCAGCAAGTACATCATCAGCTGGTCAATCA

**HRV16_5UTR.ab1_(a)**

CGTTATCCGCAAGATGCCTACGCAAAGCCTAGTAATACATTGAAAGATACTTGGTTGGTCGCTCAGCTGTTAACCCAACAGTAGACCTGGTAGATGAGGCTAGAGATTCCCCTCCGGCGACGGAGTTCTAGCCTGCGTGGCTGCCTGCACACCCACTGGGTGTGAAGCCAAGTATTGGACAAGGTGTGAAGAGCCGCGTGTGCTCATCTTGAGTCCTCCGGCCCCTGAATGTGGCTAACCTTAAACCTGCAGCCAGTGCACACAATCCAGTGTGTAGCTGGTCGTAATGAGCAATTGCGGGATGGGACCAACTACTTTGGGTGTCCGTGTTT

**HRV16_5UTRVP2.ab1_(b)**

CGTTATCCGCAAGATGCCTACGCAAAGCCTAGTAATACATTGAAAGATACTTGGTTGGTCGCTCAGCTGTTAACCCAACAGTAGACCTGGTAGATGAGGCTAGAGATTCCCCTCCGGCGACGGAGTTCTAGCCTGCGTGGCTGCCTGCACACCCACTGGGTGTGAAGCCAAGTATTGGACAAGGTGTGAAGAGCCGCGTGTGCTCATCTTGAGTCCTCCGGCCCCTGAATGTGGCTAACCTTAAACCTGCAGCCAGTGCACACAATCCAGTGTGTAGCTGGTCGTAATGAGCAATTGCGGGATGGGACCAACTACTTTGGGTGTCCGTGTTTCACTTTTTTCCTTTTATTATTGCTTATGGTGACAATATATATAGCATATATATATTGTTATCATGGGCGCTCAAGTATCTAGACAGAATGTTGGTACGCACTCAACACAAAATATGGTGTCAAATGGATCCAGCCTCAATTATTTTAACATCAATTATTTCAAAGATGCAGCTTCCAGTGGTGCTTCTCGATTGGACTTCTCTCAAGACCCCAGTAAATTC

**HRV37_5UTR.ab1_(a)**

GCCTTTACCGTTATCCGCCAACCAACTACGTAAAAGCTAGTATCATCATGTTTTAAAATAGGCGTTCGATCAGGTGGATCCCCCCTCCACTAGTTTGGTCGATGAGGCTAGGAACTCCCCACGGGTGACCGTGTCCTAGCCTGCGTGGCGGCCAACCCAGCTTCTGCTGGGACGCCTTTTTATGGACATGGTGTGAAGACTCGCATGTGCTTGGTTGTGACTCCTCCGGCCCCTGAATGCGGCTAACCTTAACCCCGGAGCCCTGTGTTGCAATCCAGTAACATTAGGGTCGTAATGAGCAATTTCGGGACGGGACCGACTACTTTGGGTGTCCGTGTT

**HRV37_5UTRVP2.ab1_(b)**

GCCTTTACCGTTATCCGCCAACCAACTACGTAAAAGCTAGTATCATCATGTTTTAAAATAGGCGTTCGATCAGGTGGATCCCCCCTCCACTAGTTTGGTCGATGAGGCTAGGAACTCCCCACGGGTGACCGTGTCCTAGCCTGCGTGGCGGCCAACCCAGCTTCTGCTGGGACGCCTTTTTATGGACATGGTGTGAAGACTCGCATGTGCTTGGTTGTGACTCCTCCGGCCCCTGAATGCGGCTAACCTTAACCCCGGAGCCCTGTGTTGCAATCCAGTAACATTAGGGTCGTAATGAGCAATTTCGGGACGGGACCGACTACTTTGGGTGTCCGTGTTTCTCATTTTTCTTATTATTGTCTTATGGTCACAGCATATATATAACGTATATACTGTGATCATGGGCGCTCAAGTGTCAACACAAAAGAGTGGATCTCACGAAAATCAAAACATACTCACCAATGGATCAAACCAGACCTTTACGGTCATAAATTACTACAAAGATGCAGCTAGTTCATCCTCGGCTAGCCAATCTTTCTCAATGGACCCATCAAAATTTACAGAACCAGTTAAAGACTTGATGTTAAAGGGAGCACCCGCATTGAACTCACCAAATGTTGAGGCTTGTGGTTATAGTGATAGAGTCCAGCAGATTACCCTAGGTAATTCAACCATAACAACCCAGGAAGCAGCCCATGCAGTTGTATGTTACGCCGAA

**CVA21_VP12A.ab1**

GGTGAGAACACCGATGCTGGCGACACGTTTTACGGTTTAGTGTCCATAAATGATTTTGGAGTTTTAGCAGTTAGAGCAGTAAACCGCAGTAATCCACATACAATACACACATCTGTGAGAGTGTACATGAAACCAAAACACATTCGGTGTTGGTGCCCCAGACCTCCTCGAGCTGTATTATACAGGGGAGAGGGAGTGGACATGATATCCAGTGCAATTCTACCTCTGGCCAAGGTAGACTCAATTACCACTTTTGGGTTTGGTCATCAGAACAAAGCAGTGTACGTTGCCGGTTACAAGATTTGCAACTACCACCTAGCAACCCCAAGTGATCACTTGAATGCAATTAGTATGTTATGGGACAGGGATTTAATGGTGGTGGAATCTAGAGCCCAGGGAACTGATACCATCGCCAGATGTAGTTGCAGGTGTGGAGTTTACTATTGTGAATCTAGGAGGAAGTACTACCCTGTCACTTTTACTGGCCCAACGTTTCGATTCATGGAAGCAAACGACTACTATC

**CVB3_VP12A.ab1**

TGGGCACGCTATATGCAAGACATGTCAACGCTGGAAGCACGGGTCCAATAAAAAGCACCATTAGAATCTACTTCAAACCGAAGCATGTCAAAGCGTGGATACCTAGACCACCTAGACTCTGCCAATACGAGAAGGCAAAGAACGTGAACTTCCAACCCAGCGGAGTTACCACTACTAGGCAAAGCATCACTACAATGACAAATACGGGCGCATTTGGACAACAATCAGGGGCAGTGTATGTGGGGAACTACAGGGTAGTAAATAGACATCTAGCTACCAGTGCTGACTGGCAAAACTGTGTGTGGGAAAGTTACAACAGAGACCTCTTAGTGAGCACGACCACAGCACATGGATGTGATATTATAGCCAGATGTCAGTGCACAACGGGAGTGTACTTTTGTGCGTCCAAAAACAAGCACTACCCAATTTCGTTTGAAGGACCAGGTCTAGTA

**CVB4_VP12A.ab1**

TGGGGACCATATATGCGCGCCATGTTAATGATTCTAGCCCAGGGGGACTGACCAGCACCATCCGCATCTACTTCAAACCCAAACACGTCAAAGCATATGTGCCACGCCCCCCCCGTTTGTGTCAATACAAGAAAGCCAAGAATGTGAACTTTGATGTTGAGGCCGTTACAGCGGAGCGTGCAAGCTTGATAACCACAGGCCCCTATGGACATCAATCAGGGGCCGTGTATGTGGGCAATTACAAGGTAGTCAATAGGCACTTGGCCACGCACGTGGATTGGCAAAATTGCGTGTGGGAGGATTATAATAGAGACCTTCTAGTGAGTACTACCACGGCCCACGGGTGCGACACCATTGCCAGATGCCAATGCACAACAGGTGTGTACTTTTGCGCCTCCAAGAGCAAACACTACCCAGTTAGCTTTGAAGGACCAGGTTTGGTGGAAGTCCAAGAAAGTGAATATTACCCAAAAAGAATACCA
