# Supplementary material for: An RNA replication-center assay for high content image-based quantifications of human rhinovirus and coxsackievirus infections
Source: Virol J. 2010 Oct 11;7:264. doi: 10.1186/1743-422X-7-264 (PMC2958916; doi:10.1186/1743-422X-7-264)
Supplement: Additional file 8 — Supplemental references. [file 1743-422X-7-264-S8.DOC]

**Additional file 8, supplemental references**

1. Lee WM, Grindle K, Pappas T, Marshall DJ, Moser MJ, Beaty EL, Shult PA, Prudent JR, Gern JE: **High-throughput, sensitive, and accurate multiplex PCR-microsphere flow cytometry system for large-scale comprehensive detection of respiratory viruses.** *J Clin Microbiol* 2007, **45:**2626-2634.

2. Hyypia T, Auvinen P, Maaronen M: **Polymerase chain reaction for human picornaviruses.** *J Gen Virol* 1989, **70 ( Pt 12):**3261-3268.

3. Jokela P, Joki-Korpela P, Maaronen M, Glumoff V, Hyypia T: **Detection of human picornaviruses by multiplex reverse transcription-PCR and liquid hybridization.** *J Clin Microbiol* 2005, **43:**1239-1245.

4. Kotla S, Peng T, Bumgarner RE, Gustin KE: **Attenuation of the type I interferon response in cells infected with human rhinovirus.** *Virology* 2008, **374:**399-410.
